# Structure and function of skin barrier lipids: Effects of hydration and natural moisturizers *in vitro*

**DOI:** 10.1016/j.bpj.2024.10.006

**Published:** 2024-10-10

**Authors:** Irene Sagrafena, Maxim Morin, Georgios Paraskevopoulos, Emelie J. Nilsson, Iva Hrdinová, Andrej Kováčik, Sebastian Björklund, Kateřina Vávrová

**Affiliations:** 1 Skin Barrier Research Group, Faculty of Pharmacy in Hradec Králové, Charles University, Hradec Králové, Czech Republic; 2 Biofilms Research Center for Biointerfaces, Malmö University, Malmö, Sweden; 3 Department of Biomedical Science, Faculty of Health and Society, Malmö University, Malmö, Sweden

## Abstract

Lipid membranes play a crucial role in regulating the body’s water balance by adjusting their properties in response to hydration. The intercellular lipid matrix of the stratum corneum (SC), the outermost skin layer, serves as the body’s primary defense against environmental factors. Osmolytes, including urocanic acid (UCA) and glycerol, are key components of the natural moisturizing factor that help the SC resist osmotic stress from dry environments. This study examines the effects of UCA and glycerol (each at 5 mol %) on isolated human SC lipids. For this, different techniques were employed, offering complementary information of the system’s multiscale characteristics, including humidity-scanning quartz crystal microbalance with dissipation monitoring, infrared spectroscopy, x-ray diffraction, electrical impedance spectroscopy, and studies of water loss and permeability. Our results show that UCA increases water sorption and makes lipid films more liquid-like at high relative humidity, without significantly altering the lipid lamellar structure, chain order, or orthorhombic chain packing. Lipid films containing UCA exhibited higher water loss and significantly higher model drug permeability compared to lipid films without UCA. Further, incorporation of UCA resulted in kinetically faster changes in electrical properties upon contact with aqueous solution compared with control lipids. These observations suggest that UCA reduces lipid cohesion in regions other than the acyl chain-rich leaflets, which may impact SC desquamation. In contrast, glycerol did not influence the hydration or permeability of the SC lipid matrix. However, it increased the proportion of orthorhombic domains at high humidities and slowed the kinetics of the hydration process, as evidenced by slower changes in the dielectric properties of the lipid film. These findings suggest that glycerol enhances lipid cohesion rather than increasing water uptake, which is typically the expected function of humectants. Consequently, UCA and glycerol appear to have distinct roles in maintaining epidermal homeostasis.

## Significance

This study investigates the effects of two natural compounds, urocanic acid (UCA) and glycerol, on the lipid matrix of the skin’s outermost layer, the stratum corneum (SC), which serves as a barrier against environmental factors. Our experiments show that UCA increases lipid hydration and makes the lipid matrix more fluid, potentially facilitating the natural shedding of dead skin cells. In contrast, glycerol reinforces the lipid matrix’s structure without altering its water sorption capacity. These findings improve our understanding of skin barrier maintenance, which is important for developing skincare treatments.

## Introduction

Water balance is a key factor in the adaptation of life, and the lipid membranes that compartmentalize, protect, and regulate the exchange of water and molecules are the central structures of this adaptation (1). In this respect, the extracellular lipid matrix in the uppermost layer of the epidermis, the stratum corneum (SC), is crucial for limiting the water loss from the body, as well as protecting it from adverse effects of the external environment. The lipid matrix has a unique multilamellar lipid structure comprised of a mixture of ceramides, cholesterol, and free fatty acids, which are minimally hydrated (2,3,4). By responding to external conditions, SC lipids effectively regulate transepidermal water loss to limit dehydration and ensure physiological conditions in dry or cold climates (5).

The general mechanism by which lipid membranes regulate the water balance of organisms relies on the fact that water controls the properties and phase behavior of lipid membranes (1,6). Thus, even the molecular properties of SC barrier lipids, although very rigid and poorly hydrated, have been shown to depend on the hydration degree (7,8). Furthermore, hydration regulates the permeability across the skin barrier for both hydrophilic and hydrophobic molecules (9,10) and is also important for the flexibility, softness, and pliability of the SC (11,12). Similarly, several studies have emphasized the importance of sufficient water levels for biochemical and enzymatic reactions to take place in the skin barrier, which is most likely crucial for maintaining a healthy status of the skin (13,14,15). Indeed, reduced SC hydration is associated with skin diseases such as atopic dermatitis (16) and psoriasis (17).

The presence of small water-soluble substances in relatively high concentrations is a common property for organisms exposed to osmotic stress, such as dry conditions (18). The outermost SC is notably exposed to a rather arid external environment and the presence of osmolytes in the SC is therefore expected. In the field of skin cosmetics and dermatology, the osmolytes are referred to as the natural moisturizing factor (NMF), and these components are known to be beneficial for skin suffering from dry conditions (19). The NMF substances contribute to the epidermal homeostasis and this effect is usually associated with their water-binding capacity (20). However, it should be noted that water and NMF can affect the physical state of the SC molecular components in a complex manner, which is why it is important to consider the combined effect of both water and the NMF (21,22). For example, the effects of NMF on water binding in the SC vary by depth, with limited binding at the surface due to folded keratin, maximal binding and swelling in the intermediate layers due to unfolded keratin, and minimal swelling at the deeper layers where water binding sites are saturated (23). In addition, some of these effects are dependent on relative humidity (RH); as Vyumvuhore et al. observed, beyond 60% RH, excess unbound water can disrupt lipid and protein structures, underscoring the critical role of hydration in maintaining the SC barrier function (24).

**Figure 1:**
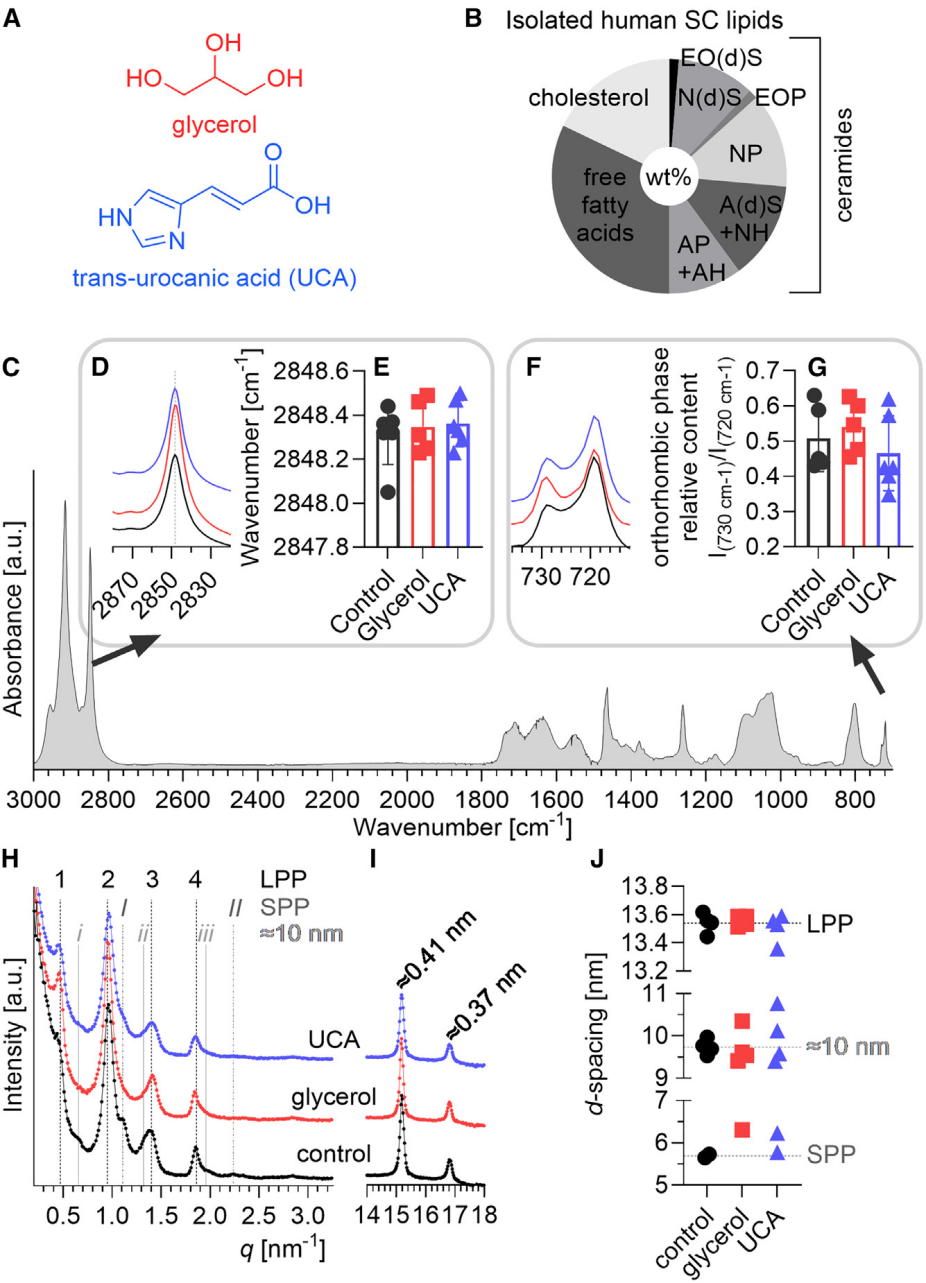
Structures of glycerol and UCA (*a*), the composition of the isolated human SC lipids by weight (*b*), FTIR (*c–g*), and SAXD and WAXD (*h–j*) results at low RH and 32°C. (*c*) Is an example FTIR spectrum of the lipids, with insets showing the effects of glycerol and UCA on lipid chain order probed by the methylene symmetric stretching band (*d–e*) and chain packing deduced from the ratio of intensities of the higher and lower wavenumber components of the methylene rocking vibration (*f* and *g*). Data are shown as individual points, *n* ≥ 4; spectra are representative. Ceramide nomenclature in (*b*) is a combination of letters defining the sphingoid base (sphingosine, S; dihydrosphingosine, dS; phytosphingosine, P; 6-hydroxysphingosine, H) and the *N*-acyl chain (unsubstituted, N; α-hydroxylated, A; ω-acyloxy substituted, EO).

Most components of NMF are formed by the degradation and processing of the intracellular histidine-rich protein filaggrin in response to the water gradient in the SC (25), for example, pyrrolidonecarboxylic acid, *trans*-urocanic acid (UCA) (Fig. 1 *a*), and free amino acids. Although filaggrin degradation products are produced intracellularly, it may be expected that these small and polar substances can, to some extent, be distributed into the polar headgroup regions of the intercellular lipid matrix and thereby influence its physical state and/or hydration degree. This may be particularly true for UCA, which is somewhat less polar than other NMF components (distribution coefficient, a pH-dependent measure of a molecule’s lipophilicity, logD at pH 5 is approximately −1) and has an affinity for lipid membranes (26). In addition, UCA is distributed systemically in the body and is particularly abundant in the SC (in the *cis*-UCA form (27)), which necessarily implies permeation through the SC intercellular lipid matrix. A previous study showed that UCA affects not only the molecular mobility of keratin in the SC cells, but also the mobility of molecular segments belonging to ceramides, fatty acids, and cholesterol in porcine SC (21).

Another important NMF component in SC is glycerol (Fig. 1 *a*). Glycerol enters the SC via the water/glycerol transporter aquaporin-3 in basal keratinocytes (28) and also from the skin surface, where it is produced by hydrolysis of triglycerides from sebaceous glands. Aquaporin-3-deficient mice have reduced SC water and glycerol levels, leading to lower skin elasticity, and impaired barrier recovery after SC removal. Interestingly, these abnormalities are corrected by glycerol administration (29) and not by SC hydration. Similarly, asebia J1 mice showed epidermal hyperplasia, inflammation, and decreased SC hydration, which can be repaired by topical glycerol but not by urea, another potent endogenous humectant (30). The safety assessment of glycerol was described in a recent review (31).

These studies suggest a more complex role for glycerol than that of a mere moisturizer. In lipid models, glycerol inhibited the phase transition from liquid crystalline to the solid phase in a dry atmosphere (32). Similarly, glycerol (32) stabilized the liquid crystalline phase of dimyristoyl phosphatidylcholine at low humidities, which otherwise would induce a solid gel phase (33). Glycerol further prevented major changes in pig skin permeability caused by dehydration (34) and increased the mobility of both keratin and the lipid chains similarly compared with hydration, but only marginally influenced the SC water content (21). Based on these studies, it is likely that glycerol may substitute water rather than attracting it by providing similar hydrogen bonding to lipid polar headgroups (33).

Given the broad use of glycerol in topical products and the implication of both glycerol and UCA in diseases, it is mandatory to better understand their effect on the skin, including the extracellular lipid barrier. In this study, we investigate the effects of 5 mol % glycerol and 5 mol % UCA on isolated human SC lipids, eliminating the confounding effects due to hydration of the protein components of the SC cells, i.e., the corneocytes. We employ a combination of techniques to probe different aspects of the SC lipid lamellar matrix, offering complementary insights into how glycerol and UCA affect both the macroscopic properties and the molecular structure of the lipid film. Specifically, humidity scanning quartz crystal microbalance with dissipation monitoring (HS QCM-D) provides water sorption isotherms and examines how hydration influences the viscoelastic properties of lipids on a macroscopic level. However, HS QCM-D is less sensitive to macroscopic defects in the lipid films, which is why we also employ electrical impedance spectroscopy (EIS), water loss, and permeability experiments to provide information on the macroscopic barrier properties. Finally, Fourier transform infrared spectroscopy (FTIR) and small- and wide-angle x-ray diffraction (SAXD and WAXD) offer molecular insights into the lipid chain order, lateral packing, and organization of lamellar phases. This complementary approach allows us to conclude that UCA increases water sorption and permeability properties of the lipid film, making it more liquid-like, without significantly altering the molecular properties of the lipid lamellae. Conversely, glycerol enhances lipid cohesion, increases the proportion of orthorhombic domains, and slows the hydration process, without affecting the hydration or permeability characteristics of the lipid film. Thus, this study indicates distinct effects of UCA and glycerol on the SC lipid matrix.

## Materials and methods

### Materials

Silica gel 60 (230–400 mesh) for column chromatography, thin-layer chromatographic (TLC) plates (silica gel 60 F 254, aluminum back), high-performance TLC (HPTLC) glass plates (silica gel 60; 20 × 10 cm and 10 × 10 cm), gentamicin sulfate from *Micromonospora purpurea* (700 U/mg), trypsin from porcine pancreas (1500 U/mg), glycerol, indomethacin, sodium phosphate dibasic dodecahydrate, propylene glycol, solvents (HPLC grade) and buffer components were purchased from Merck (Darmstadt, Germany). *trans*-UCA was supplied by TCI (Tokyo, Japan). Nylon membranes (pore size 0.45 *μ*m, diameter 47 mm) were supplied by Fisher Scientific (Pardubice, Czech Republic). All chemicals were of analytical grade and were used without further purification. All aqueous solutions were prepared using Millipore water (Milli-Q system, Millipore, Burlington, MA). Esco microscope cover glasses 22 × 22 mm^2^ were obtained from Erie Scientific (Portsmouth, NH). Nuclepore track-etched polycarbonate membranes (0.015 *μ*m pore size) were purchased from Whatman (Maidstone, UK). AT-cut SiO_2_ sensors (QSX 303, 5 MHz) were from Biolin Scientific AB (Gothenburg, Sweden). DuPont Kapton with 0.013 mm thickness was obtained from Goodfellow Cambridge (Huntingdon, England).

### Human SC lipids

Human skin was obtained from healthy female donors who underwent plastic surgery and beforehand had signed a written informed consent. The process was approved by the Ethics Committee of the Sanus First Private Surgical Centre (Hradec Králové, Czech Republic, no. 03/11/22) and was carried out in accordance with the principles of the Declaration of Helsinki. The SC was isolated using trypsin, the lipids extracted using chloroform/methanol mixtures, and purified by column chromatography using a protocol previously described in (35). HPTLC was used to check both the purity and proportions within the isolated human SC lipids subclasses (35,36).

### Preparation of SC lipid models with or without NMF

The concentrations of glycerol and UCA in the SC are approximately 0.8 mmol of glycerol per gram of SC protein (30) and 0.6 mmol of UCA per gram of SC protein (37). Given their largely hydrophilic nature, with logP (log of the partition coefficient of a solute between octanol and water, the basic measure of lipophilicity) around −1.8 for glycerol and −1.0 for UCA at pH 5 (26), it is reasonable to assume that they will primarily be found in the corneocytes, the SC cells. Still, it is likely that these substances will also partition to some extent into the extracellular lipid matrix of the SC. In a pilot study, we first tried to incorporate 5, 10, and 50 mol % of these substances NMF into the lipids of the SC to better see any NMF effects. However, confocal Raman microspectroscopy showed phase separated UCA already at 10 mol % (data not shown). Therefore, we chose 5 mol % NMF in the lipid model for this study, which corresponds to approximately 1 wt % glycerol and 1.5 wt % UCA. These concentrations are also plausible to achieve after topical application of skin care products containing these substances.

Control models contained only isolated human SC lipids, while the NMF-containing models incorporated 5 mol % of either glycerol or UCA into the lipids. The total amount of lipids remained the same for all samples. Lipid solutions were prepared at 1.25 mg/mL in hexane/ethanol 96% 2:1 (v/v); glycerol and UCA were added to lipids at 5 mol % from stock solutions in 96% ethanol. The lipid solutions with or without NMF were sprayed (2 × 100 *μ*L per 1 cm^2^) using the Linomat V (Camag, Muttenz, Switzerland) (36) under a stream of nitrogen on either glass coverslips (for SAXD, WAXD, and FTIR experiments) or nucleopore polycarbonate filters with 0.015 *μ*m pore size (for EIS and permeation experiment), resulting in lipid films of about 2.5 *μ*m in thickness. For the HS QCM-D experiment, lipids were sprayed to AT-cut SiO_2_ sensors at a concentration of 0.25 mg/mL (≈500 nm thickness) to match the sample thickness requirements of this technique (38). Under spray conditions, the organic solvents evaporated, leaving a film of lipids. Overnight vacuum was used to remove residual solvents. The models were annealed at 70°C in a water vapor-saturated chamber for 20 min and, after that, allowed to cool over 4 h.

### SAXD and WAXD

#### Samples at 25–90% RH

SAXD and WAXD were measured in a humidity chamber on a Xeuss 3.0 instrument (Xenocs, Grenoble, France), with an x--ray beam generated by a CuKα radiation source (λ = 1.542 Å). The samples were scraped off the support base, mounted on Kapton films, and placed in a chamber with controlled temperature and humidity. The measurements were performed at 32°C and three different RHs: 25, 70, and 90%. The detector was a Pilatus3 R 300K hybrid photon counting detector with a sample-to-detector distance (STDD) of 600 mm (SAXD) and 285 mm (WAXD). The samples were measured for 30 min at each relative humidity and STDD. These two STDDs covered the q-range 0.01 ≤ *q* (nm^−1^) ≤ 18, where *q* is the scattering vector defined as |***q***| = *q* = 4*π*/*λ* sin (*θ*/2) and θ is the scattering angle. The 1D diffraction curves were obtained by azimuthal integration of the 2D pattern, corrected for background scattering and normalized to the direct beam, using the Xenocs XSACT software (version 2.6). The q-scale was calibrated using silver behenate. Data evaluation was performed using MATLAB R2021a with background subtraction, linear regression, and peak fitting with a single Gaussian curve.

#### Samples at ∼99% RH

These measurements were performed as above at 32°C but using a multipurpose Peltier gel-holder stage. The lipid models on Kapton film were exposed to a drop of PBS (pH 7.4) with water activity of approximately 0.99 for a duration of 4 h (34). Then, the excess buffer was removed and the Kapton with the hydrated lipids was mounted onto a holder for gels (eight positions) and sealed with another empty Kapton disc using an O-ring spacer. Data evaluation was performed using MATLAB R2021a with background subtraction, linear regression, and peak fitting with a single Gaussian curve.

### FTIR

Infrared spectra were collected using a Nicolet 6700 spectrometer (Thermo Fisher Scientific, Waltham, MA) with a single-reflection MIRacle attenuated total reflection ZnSe crystal (PIKE Technologies, Madison, WI,). The spectra were collected in a closed chamber either at room temperature or while gradually increasing the temperature in 2°C steps in 15-min intervals at a resolution of 2 cm^−1^. The final spectra were generated by the coaddition of 256 scans.

### HS QCM-D

Water sorption isotherms of thin films of extracted human SC lipids were determined by HS QCM-D (6,38). In brief, the QCM-D technique works by exciting a piezoelectric quartz sensor into resonance by applying an alternating voltage, while the frequency of the resulting oscillating shear motion is monitored. Any mass adsorbed or desorbed from the sensor results in a change of the frequency and this parameter can therefore be used to accurately determine the mass coupled to the sensor surface. The mass of the adsorbed material can be determined by the Sauerbrey equation (Δm=−Δf×C) as long as the adsorbed mass is small compared with the mass of the crystal and the material is rigidly adsorbed and homogenously distributed over the active area of the crystal (39). The Sauerbrey equation describes the relationship between the negative frequency change normalized per overtone, *Δf*, and the change of the areal mass of the adsorbed material, *Δm*, where *C* is a constant that depends only on the intrinsic properties and the thickness of the quartz disc (40). In addition to *Δf*, the viscoelastic properties of the adsorbed material are probed via the changes of the dissipation data, *ΔD*, which is related to the change in the decay time of the oscillating resonator when the alternating potential is turned off (40).

A Q-sense E4 instrument, equipped with the humidity module QHM 401 and AT-cut SiO_2_ (QSX 303, 5 MHz) sensors, was used in this work (Biolin Scientific AB, Gothenburg, Sweden). The general experimental procedures of the humidity scanning experiment are described in detail elsewhere (6,38). In brief, baselines of uncoated sensors were measured in a dry N_2_ atmosphere at 25°C. Next, the lipid films were deposited onto the sensor as described above. Subsequently, the coated sensor was dried in the humidity module under the flow of N_2_ gas until a stable baseline of the frequency was obtained. Next, the humidity scanning experiment was initiated by flowing a LiCl solution with defined water activity (*a*_w_) through the humidity module. Since only water vapor can pass across the Gore membrane of the humidity module, the relative humidity above the sensor is regulated by continuously adjusting the *a*_w_ of the LiCl solution, from 11% up to ∼100% RH (*a*_w_ = RH/100%). The mass of the dry lipid film, as well as the amount of water taken up by the lipid film during the water sorption experiments, was calculated according to the Sauerbrey equation. The film thicknesses (*h*) of the dry lipid film were estimated from the areal mass of the dry film according to ${h=m}_{\text{dry}}/\rho$, assuming a lipid density equal to be ρ = 0.87 g/cm^3^ (41). The *Δf* and *ΔD* were primarily evaluated based on overtones three and five by using MATLAB R2021a.

### Water loss and permeability

The model lipid films on Nuclepore filter support were sandwiched between Teflon holders with a 0.5 cm^2^ circular opening. Then, the holders were mounted between the donor and the acceptor parts of Franz diffusion cells with an approximate acceptor phase of 7 mL filled with PBS buffer (pH 7.4) containing 50 mg/L gentamicin. The specific volume of the acceptor phase was measured and used for data calculations. Once the Franz cells were fully mounted, they were placed in the water bath at 32°C and left to equilibrate for 12 h. The next day, water loss was measured at least 2 times using an AquaFlux AF 200 instrument (Biox Systems, London, UK), with the condenser-chamber measurement method. The cells were then left to equilibrate overnight. The day after, 100 *μ*L of 2% indomethacin suspension in PBS was applied to the donor section of the Franz cells, onto the membranes. A total of 300 *μ*L were withdrawn from the acceptor phase every 2 h over 10 h and at the same time were replaced by the same volume of fresh PBS. The concentration of indomethacin in the acceptor phase was detected using a previously published HPLC method (42).

### EIS

Nuclepore-supported lipid films were clamped between the donor and acceptor chambers of conventional Franz cells with a 9 mm orifice. However, the surface area of the lipid film being probed by the EIS was adjusted to 0.30 cm^2^ by inserting silicone rings in each chamber. A circulating water bath kept the temperature of the Franz cell stable at 32°C for the duration of the experiment. Both the donor and acceptor phases were filled with PBS buffer (pH 7.4). Measurements were performed for up to 4 h in the following manner: scans 1–11 were performed with 60 s in between each measurement (from 0 to 10 min), every 5 min from scans 11 to–17 (from 10 to 40 min), with 10-min intervals between scans 17 and 20 (from 40 to 70 min), after 20 min was obtained scan 21 (90 min), after half an hour from scan 21, measurement 22 (2 h) was recorded, and finally scans 22–24 with 60-min intervals (from 2 to 4 h), which corresponded to a total time of 4 h. The impedance data were modeled with an electrical circuit consisting of a resistor (solution resistance, *R*_sol_) in series with a parallel arrangement of a resistor (membrane resistance, *R*_mem_) and a constant phase element (43,44). The constant phase element is an empirical element that accounts for the nonlinear distribution of time constants and can be utilized to determine the effective capacitance of the investigated membrane (45). The method used for analyzing the impedance data is described elsewhere (44). Data evaluation was performed using MATLAB R2021a.

### Statistical analysis

One-way ANOVA with Tukey’s multiple comparisons test was used to analyze three or more groups (GraphPad Prism version 8.2.1, GraphPad Software, Boston, MA). All data are presented as the mean and standard deviation (SD); with the specific number of replicates (*n*) in each figure. A *p* value <0.05 was considered statistically significant.

## Results

### At low humidity, neither glycerol nor UCA significantly alters the lamellar and lateral arrangement of human SC lipids

To study the specific interactions of NMF with skin barrier lipids, we used lipids isolated from human SC and chromatographically purified to remove surface lipids or triglyceride contamination that may have occurred during tissue processing. HPTLC confirmed the presence of all subclasses of barrier lipids (Fig. 1 *b*). Based on this analysis and the chain length distribution of lipids from the literature (46), we approximated the average molar mass of these lipids to be 475 g/mol.

The models prepared from these isolated SC lipids, without added UCA or glycerol, have predominantly well ordered, tightly packed chains, as inferred by FTIR. In particular, the methylene stretching at 2848.3 ± 0.3 cm^−1^ suggests predominantly all*-trans* chains (Fig. 1, *c*–*e*) and the rocking band doublet at 730 and 720 cm^−1^ indicates orthorhombic chain packing (Fig. 1, *f* and *g*). In addition, these lipids are organized into a long periodicity lamellar phase (LPP) with a repeat distance, *d* = 13.5 ± 0.1 nm (Fig. 1, *h* and *j*). In two out of four of the reference samples, one weak reflection, corresponding to *d* = 5.7 ± 0.1 nm, which can be assigned to a short periodicity phase (SPP), was also detected. All the diffractograms also had a very weak reflection at *q* ∼0.65 nm^−1^ corresponding to a *d* = 9.7 ± 1.8 nm. Separated cholesterol was not visible, but it is possible that its first reflection overlapped with the fourth reflection of the LPP. In the wide-angle region, two reflections at *q* = 15.2 and 16.8 nm^−1^ (Fig. 1 *i*), corresponding to distances between diffracting planes ∼0.41 and ∼0.37 nm, respectively, and confirmed the presence of orthorhombically packed lipid chains, which is typical for the lipids of healthy human SC (47).

Next, we incorporated glycerol and UCA into the SC lipids. At low humidity, no significant effects of glycerol or UCA on lamellar or lateral lipid organization were observed (Fig. 1). Interestingly, the reflection attributable to SPP was only found in one out of four samples containing glycerol and its estimated *d* was 6.3 nm, which is higher than that in control or UCA samples (although this must be treated with caution as only one reflection was found). FTIR revealed a trend to less orthorhombically packed lipids in the presence of UCA compared with control (but not statistically significant).

### At high humidity, UCA, but not glycerol, increases water sorption and alters the viscoelastic properties of the human SC lipids

**Figure 2:**
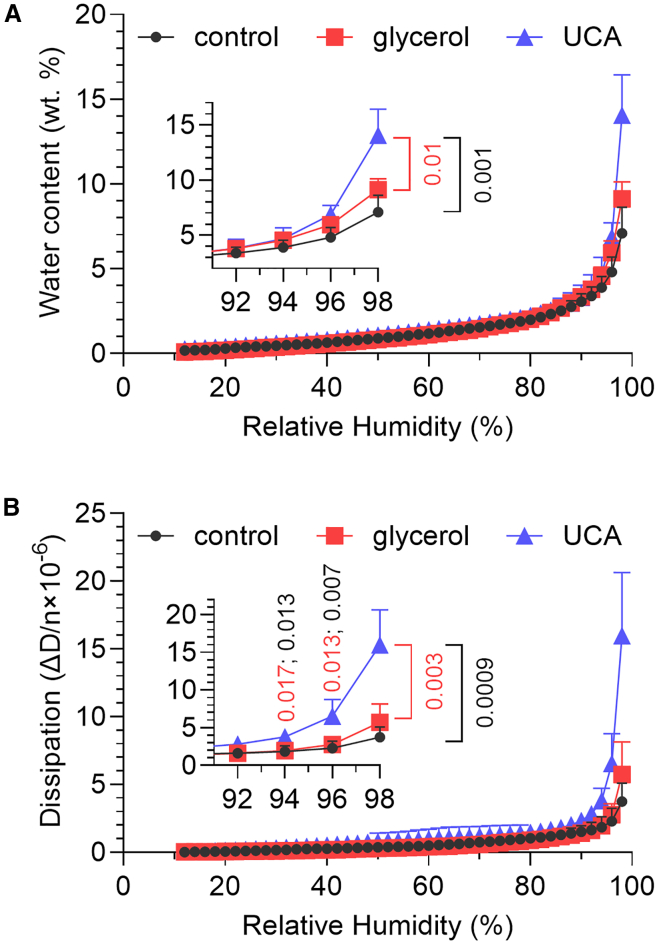
Effects of glycerol and UCA on water sorption (*a*) and dissipation (*b*) of thin (≈500 nm) human SC lipid films studied by HS QCM-D. Mean ± SD (*n* = 3–4), *p* values are given for statistically significant differences between UCA and control samples (values in *black*) and UCA versus glycerol samples (values in *red*).

HS QCM-D is a technique that provides water sorption isotherms of relatively thin films of various samples (38). Still, it is important to underline that HS QCM-D provides water sorption isotherms equivalent to the corresponding isotherms obtained with bulk samples (6,48). Thus, this method probes the hydration process of the SC lipid matrix on a macroscopic scale. The results show that the SC lipids only take up water to a very limited extent (about 7 wt %) and only at high environmental humidity, at about 98% RH (Fig. 2 *a*). This value roughly corresponds to 2 water molecules per lipid, which agrees well with the literature (41). This behavior did not change significantly in the presence of 5 mol % glycerol (water content was slightly but not significantly increased compared with lipids alone, *p* = 0.24). However, the presence of 5% UCA in the lipids increased the water sorption at 98% RH approximately twofold.

The dissipation data offer insights into the overall viscoelastic behavior of the SC lipid films. The dissipation curve corresponding to the control SC lipid film increases slightly at 98% RH compared with lower RH values (Fig. 2 *b*), implying that the lipid film becomes more fluid-like upon hydration. Similar behavior (with slightly higher dissipation values compared with control) was also seen for the lipids with glycerol. In the presence of UCA and 94–98% RH, the dissipation reaches 2–4 times higher values compared with the control.

### At high humidity, UCA and glycerol modulate SC lipid phase transitions and chain packing, but not lamellar arrangement

The LPP repeat distance in the SC lipids did not change significantly upon hydration, confirming that LPP does not exhibit noticeable swelling (49). While the reflection attributed to SPP was detected in two out of four of the control samples at 25–90% RH, it was found in all four samples at 99% RH. The intensity of the weak ∼10 nm phase (reflection at *q* = 0.65 nm^−1^ normalized to the second LPP reflection) decreased linearly with hydration of the system between 25 and 90% RH but was again slightly higher in the systems prepared at 99% RH. Lipids with 5 mol % glycerol did not differ from control at 25–90% RH but at 99% RH neither the SPP nor the ∼10 nm phase were detected and the LPP reflections slightly shifted. The incorporation of 5 mol % UCA did not affect the SAXD patterns. In all samples at all RH levels, WAXD reflections at *q* = 15.2 and 16.8 nm^−1^ with similar relative intensities were found indicating that glycerol or UCA did not alter the relative proportion of the orthorhombic and hexagonal lipid packing detected by WAXD (data not shown).

**Figure 3:**
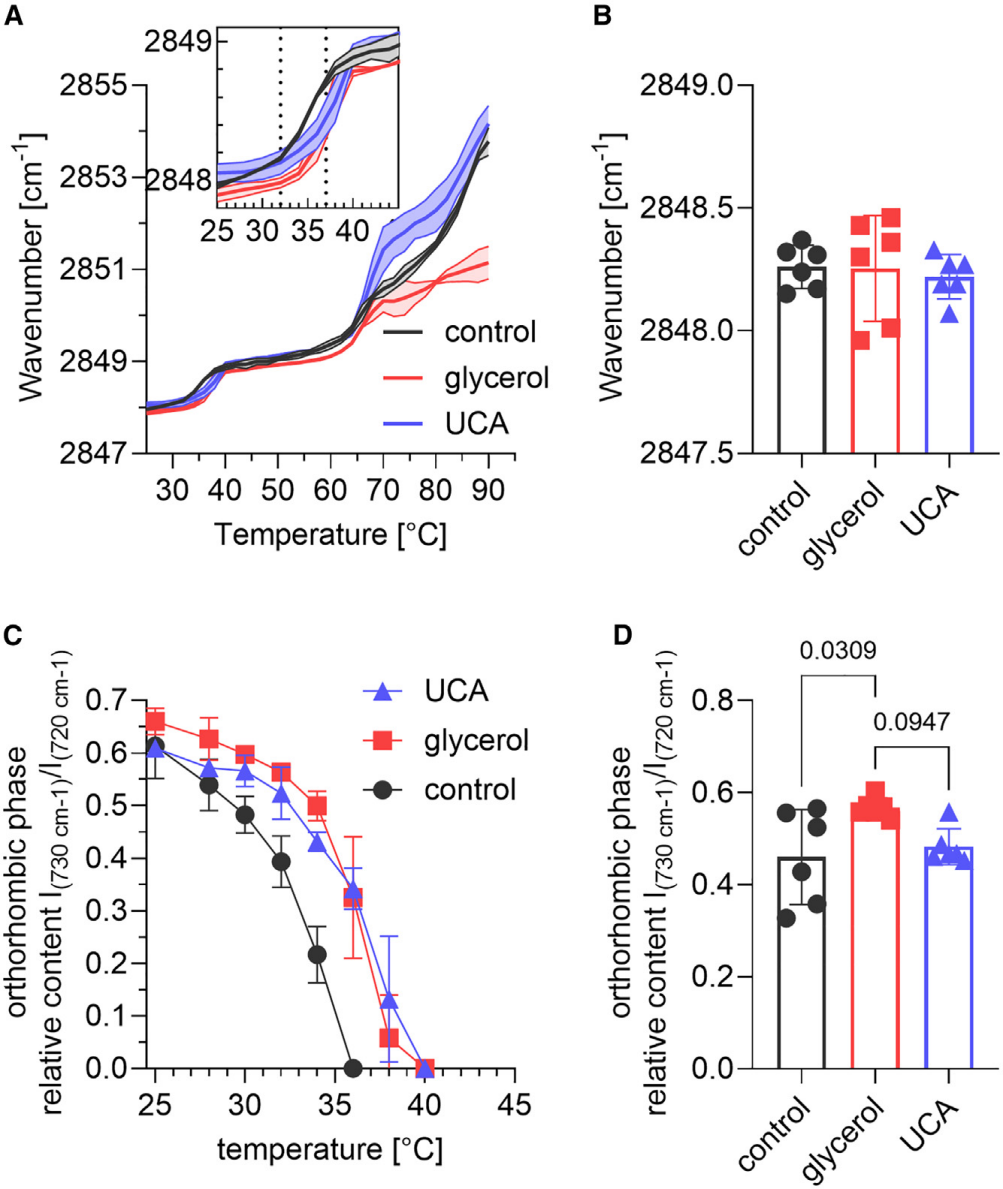
Effects of glycerol and UCA on human SC lipids probed by FTIR at ∼99% RH. Lipid chain order probed by methylene symmetric stretching wavenumber in temperature (*a*) and at 32°C (*b*). Relative content of the orthorhombic chain packing deduced from the rocking doublet in temperature (*c*) and at 32°C (*d*). Data are shown means with SD (*a* and *c*) (*n* = 2) or as individual points (*b* and *d*) (*n* ≥ 5; *p* values are indicated).

Lipid models with and without glycerol and UCA hydrated at approximately 99% RH were also studied by FTIR. Fig. 3 *a* shows the evolution of the methylene symmetric stretching on heating. Both glycerol and UCA slightly changed the pretransition temperature from about 35 to almost 40°C, but the chain order at 32°C was comparable (Fig. 3 *b*). The most pronounced differences were observed at temperatures above the main phase transition (around 65°C), where the lipids with UCA were significantly more disordered, whereas glycerol limited this temperature-induced lipid chain disordering suggesting stronger interactions. In fact, Fig. 3 *a* shows that the symmetric stretching of methylene groups remained below 2851 cm^−1^, which could be consistent with a liquid-ordered phase, even at high temperatures with glycerol. The temperature evolution of the rocking doublet (shown as the relative intensity of its 730 cm^−1^ component to the 720 cm^−1^ band in Fig. 3 *c*) confirmed the persistence of orthorhombically packed chains at higher temperatures in the presence of NMF compared with control samples, showing that UCA and glycerol have noticeable effects on the physical properties of the SC lipids. At 32°C, lipids with glycerol showed a greater relative abundance of orthorhombically arranged chains compared with the control (*p* = 0.03; Fig. 3 *d*), while this effect was not statistically significant in the case of UCA (*p* = 0.09; Fig. 3 *d*).

### UCA, but not glycerol, increases permeability of the SC lipid films

**Figure 4:**
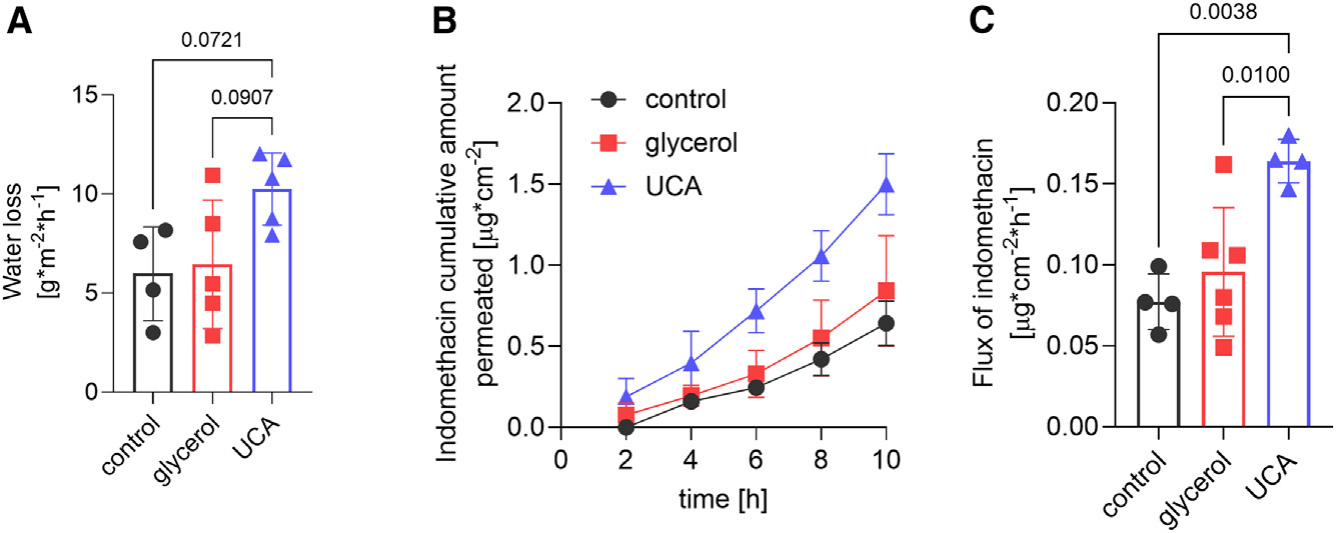
Effects of glycerol and UCA at 5 mol % on the barrier properties of human SC lipid films. Lipid films on porous support were sandwiched in Franz diffusion cells and water loss (*a*) and permeation of a model lipophilic compound indomethacin (*b* and *c*) were measured. Indomethacin permeation is shown as cumulative amounts of indomethacin permeated in time in (*b*) and calculated indomethacin flux values (from the linear portion of the permeation profile) in (*c*). Mean ± SD (*n* = 4–6), *p* values are given for relevant comparisons; in (*b*), UCA samples are significantly different from control at all time points and from glycerol at 4–10 h at *p* < 0.05.

The effects of incorporated glycerol or UCA on the functional barrier properties of SC lipids were further investigated. We assessed their effect on water loss and the permeability to a model lipophilic substance, indomethacin, using lipid films mounted in Franz diffusion cells (Fig. 4). Water loss values were not significantly different among samples, but there was a trend toward higher water loss in the presence of UCA compared with the lipid film without NMF (*p* = 0.07; Fig. 4 *a*). The indomethacin permeation increased approximately twofold in the presence of UCA compared with the control lipid sample, whereas glycerol did not induce any significant changes (Fig. 4 *a*).

### UCA causes a rapid decrease in the electrical resistance of SC lipids upon hydration

**Figure 5:**
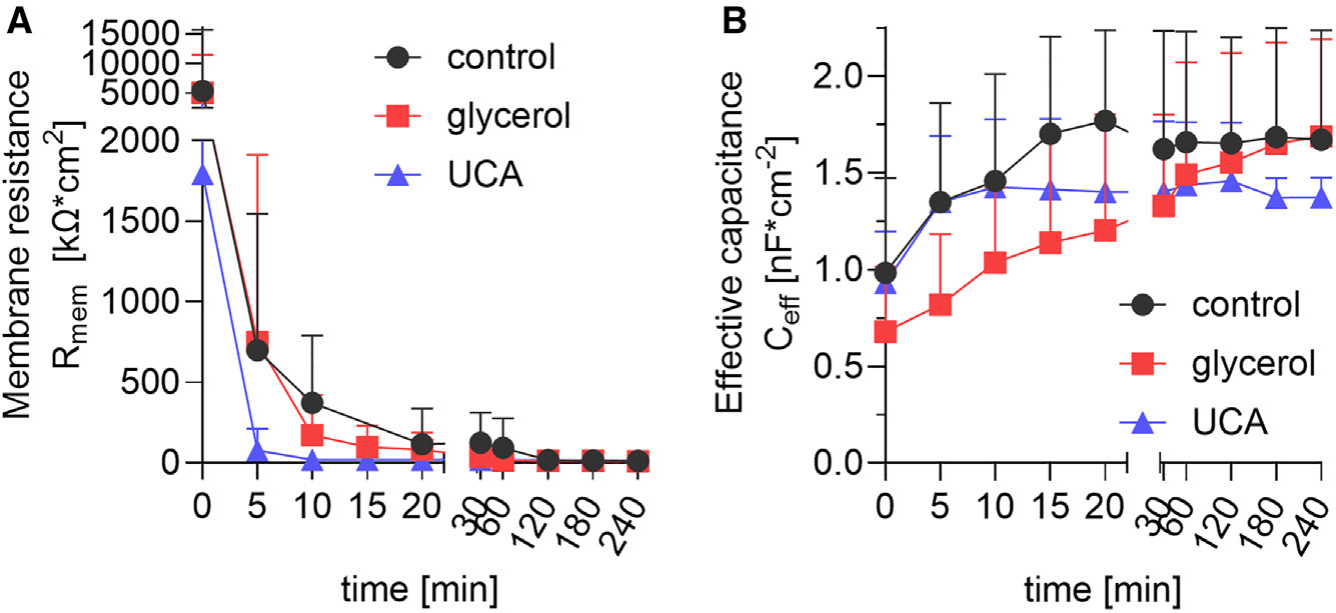
Effects of glycerol and UCA at 5 mol % on the electrical properties of human SC lipid films. Lipid films on porous support were sandwiched in Franz diffusion cells and investigated by EIS. (*a*) Shows the evolution of membrane resistance and (*b*) the evolution of effective capacitance in time. Mean ± SD (*n* = 4–6).

Human SC lipid films with/without UCA or glycerol sandwiched in Franz cells were then examined using EIS over time (Fig. 5). Similarly to the water loss and permeability experiments presented above, EIS probes the functional barrier properties on a macroscopic scale via the electrical resistance of the lipid film. The initial resistance values of the control and glycerol samples were around 5 MΩ × cm^2^ and decreased rapidly (within 15 min) to tens of kΩ × cm^2^ (Fig. 5 *a*). No differences in either absolute values or kinetics were found between lipids with and without glycerol. The resistance of the lipid films with UCA started at lower values of about 2 MΩ × cm^2^ and dropped abruptly by 96% within 5 min and remained at approximately tens of kΩ × cm^2^ for the rest of the experiment (120 min).

The second parameter that we focus on by the EIS data is the effective capacitance, which provides macroscopic scale information on the dielectric properties of the lipid film. However, the capacitive currents are obtained at significantly higher frequencies as compared with the electrical resistance and therefore not sensitive to defects of the lipid barrier. The initial values of the effective capacitance were around 1 nF/cm^2^ of the control samples and increased to approximately 1.7 nF/cm^2^ within 15 min (Fig. 5 *b*). The capacitance of the lipids films with UCA started at similar values and then leveled at around 1.4 nF/cm^2^ within 5 min. Finally, the capacitance of the glycerol samples started at 0.7 nF/cm^2^, followed by a continuous increase for the entire duration of our measurements with final values around 1.7 nF/cm^2^ after 240 min.

In the case of the lipid films, showing close to ideal capacitance properties, we can conceptualize the dielectric properties using a simple plate capacitor model with a dielectric lipid medium between the working and counter electrodes; $\varepsilon =hC_{eff}/\varepsilon_{0}$. Here, *h* is the thickness of the lipid (2.5 *μ*m), *ε*_0_ is the permittivity of vacuum (8.9 × 10^−14^ F cm^−1^), and $C_{eff}$ is obtained from the measurements (Fig. 5 *b*). As shown in this study, the lipid matrix does not swell due to hydration to any observable degree; implying that *h* can be treated as constant. Thus, based on the determined values of *C*_eff_, we obtain values of dielectric constants summarized in Table 1. The dielectric constant of the SC lipid matrix is not readily available in standard databases. However, these values are comparable with other organic molecules, which generally have dielectric constants in the range of 2–10.

**Table 1:** Estimated dielectric constant (relative permittivity, *ε*) values for the investigated human SC lipid films

	Dielectric constant, *ε*		
	Initial (0 min)	Final (240 min)	Increase (%)
Control	2.8	4.8	70
Glycerol	2.0	4.8	143
UCA	2.8	3.9	40

## Discussion

### A multiscale approach to understand how glycerol and UCA affects the structure and function of skin barrier lipids

In this study, we employed complementary techniques to explore a range of physicochemical parameters of the SC lipid matrix, with careful consideration of the system’s multiscale characteristics. It is worth noting that the x-ray diffraction and FTIR experiments focus on the molecular organization of the lipid lamellae. In particular, SAXD probes the organization of the lamellar phases at length scales of nm (i.e., SPP and LPP), whereas WAXD examines the lateral acyl packing of the lipids with Ångström resolution (i.e., orthorhombic, hexagonal, or fluid/disordered). Finally, FTIR probes the aliphatic chain bond vibrations and gives insight into the molecular dynamics of the lipids, such as lipid fluidity and lateral acyl packing. The FTIR results therefore provide complementary information to SAXD and WAXD data. Although these techniques provide understandings of the molecular properties of the lipid matrix, it is important to underline that they are insensitive to defects of the macroscopic barrier properties of the lipid films. Therefore, water loss and model drug permeability experiments and electrical resistance measurements were conducted to acquire information on the macroscopic scale, which ultimately determines the functional barrier properties. In addition, HS QCM-D measurements provide water sorption isotherms and information about the viscoelastic properties of the lipid films on a macroscopic scale (i.e., bulk properties) and is not particularly sensitive to defects within the lipid film on the molecular scale. The effective capacitance calculated based on EIS data provides insight into how the dielectric properties are affected by the NMF and can also be considered a bulk parameter, as it is not necessarily sensitive to barrier defects in the lipid film. Considering these aspects, the following discussion highlights our understanding of the structure and function of the SC lipids that emerges from this multiscale approach for studying the effects of incorporating 5 mol % of glycerol or UCA into the SC lipid matrix.

### Glycerol and UCA affect SC lipid hydration differently and also behave differently when incorporated into lipids compared with their pure forms

The first conclusion from our results is that glycerol and UCA have a minor effect on the structure and organization of the lipid matrix under dry conditions (Fig. 1). However, at high humidities, the data show that glycerol and UCA influence the properties of the extracellular lipid matrix of the human skin barrier differently. Specifically, these substances exhibit distinct behaviors when incorporated into lipids compared with their pure forms. For example, pure glycerol absorbs approximately 65 wt % water at equilibrium with 85% RH (21). However, when added to SC lipids, glycerol did not change the system’s ability to take up water (Fig. 2 *a*). This lack of increased hydration suggests that glycerol is incorporated into the lipid matrix via hydrogen bonding with the polar lipid headgroups, thereby limiting its ability to disrupt existing hydrogen bonds and form new ones with water molecules. In contrast, UCA increased the water sorption by the lipids approximately twofold at 98% RH. Notably, pure UCA absorbs significantly less water compared with glycerol, with less than 1 wt % water uptake at 85% RH (21). Therefore, this difference is likely due to UCA’s effect on the physical properties of lipids, leading to increased water uptake by the total system rather than UCA alone.

### Does UCA interact differently with the SC lipid matrix as compared with glycerol?

The observed differences in water sorption capacity of the SC lipids in the presence of glycerol and UCA should be related to the effects of these substances on the lipid chain order and lateral packing (i.e., Fig. 3). Considering that both glycerol and UCA stabilized the orthorhombic phase by shifting the transition to hexagonal chain packing to higher temperatures, it is unlikely that the water sorption is associated with alterations of the orthorhombically organized acyl chains. An alternative explanation is that glycerol and UCA interacts differently with the SC lipids, ultimately leading to different water sorption capacity of the lipid matrix. Given the distinct chemical structures of glycerol and UCA (see Fig. 1 *a*), it is logical to take this into account. For example, the carboxyl group of UCA is likely to be located in the polar head regions of the lipid matrix, while its imidazole ring has a potential to engage in π-π interactions and thus be buried deeper in the hydrophobic regions of the SC lipids. Since the incorporation of the imidazole ring between tightly arranged acyl chains would be very disadvantageous, and the relative abundance of orthorhombic lipid chains does not decrease significantly, it can be assumed that UCA is more likely to be incorporated into cholesterol-rich leaflet of the lipid matrix. Similar selective fluidization of cholesterol-rich regions has recently been described for cholesterol sulfate and ionic liquids in skin lipid models, although these were simplified with fewer lipid species than the human SC lipids used here (50,51). This reasoning might account for the distinctly different effects seen at temperatures above the main lipid phase transition, where UCA promoted heat-induced lipid disordering, while glycerol limited this effect, indicating preserved lipid order and stronger interactions. Further support comes from the dissipation data, which provide insights into the viscoelastic behavior of the SC lipid films. In other words, the selective partitioning of UCA may lead to slipping under shear stress induced by the oscillating quartz crystal, which could potentially explain the significant increase in dissipation at high hydration without any significant structural alterations. Consequently, such UCA-induced regions of a less cohesive lipid matrix could enable higher diffusion rates for water, electrolytes, and the model substance.

On the other hand, the inability of glycerol to increase water sorption by human skin lipids indicates its confinement in lipid layers and saturation of its hydrogen bonds by interactions with polar lipid heads. These hydrogen bonds may be responsible for the higher thermostability of the orthorhombic phase and the lower degree of disorder of the lipid chains after the main phase transition. Thus, glycerol appears to contribute to increased cohesion of the individual leaflets of the lipid matrix and increased stability of the orthorhombic domains without altering the structure of the LPP or the permeability of the lipid matrix, rather than acting as a humectant.

### UCA, but not glycerol, influences the functional barrier properties of SC lipids

Although UCA did not affect the overall lipid lamellar organization, its impact on the water sorption isotherm and viscoelastic properties aligns with its effects on the functional barrier properties of SC lipids. The latter was assessed by water loss, probing the inside-outside barrier, and the permeability to indomethacin, as a marker of the outside-inside barrier, indicated increased lipid permeability induced by UCA but not glycerol. Furthermore, these observations are in line with the faster drop of the electrical resistance detected in the case of UCA (Fig. 5 *a*) as compared with glycerol or control. Taken together, the increased capacity to take up water and the elevated dissipation values induced by UCA, supports the idea that UCA increases the global fluidity of the lipid system thereby making its barrier properties more permeable.

Interestingly, lipids with glycerol showed more orthorhombically arranged lipid chains than control at skin temperature (i.e., 32°C), as shown in Fig. 3 *d*. Given that orthorhombic packing is a key feature of skin lipids and is directly associated with water loss (52), it is somewhat surprising that lipids with glycerol did not show lower water loss values compared with control. In fact, the lipid samples containing glycerol showed similar macroscopic barrier properties as compared with the control samples in terms of water loss, model drug permeability, and electrical resistance.

### Insights from EIS measurements on lipid films—Eliminating the confounding effects of corneocytes, the SC cells

The ElS data obtained from the SC lipid films showed some interesting features of the electrical properties compared with what is normally observed for excised skin membranes. The initial resistance values around 2–5 MΩ × cm^2^ are significantly higher as compared with typical values obtained with excised skin membranes, ranging between 10 and 200 kΩ × cm^2^ (44,53). This may be expected given the hydrophobic character of the SC lipid matrix and its foreseen resistance to the passage of hydrophilic charge carriers. However, the abrupt decline in resistance, manifesting within minutes, is rather unexpected. Given that the structural characteristics of the lipid matrix remain largely intact despite hydration, this observation suggests the presence of global structural defects within the lipid films, facilitating the entry of water and the subsequent transport of electrolytes across the lipid film. The rather drastic drop in the resistance implies that this mechanism of water filling defective voids in the lipid films occurs within minutes. After this initial phase, the resistance values reach stable plateaus with comparable values to what is typically obtained with excised skin membranes, thereby affirming that the lipid films closely resemble the primary barrier constituent of the SC.

When comparing the kinetics of the resistance decay, the results revealed that the control and glycerol samples required about 15 min to reach stable values. For the lipid films with UCA, this process was completed in about 5 min. Hence, it is plausible that the integration of UCA into the lipid films leads to a less homogenous lipid matrix with larger or more accessible void regions, facilitating the rapid ingress of water and electrolytes. As discussed above, this is in line with the observed elevated permeability of the lipid films containing UCA toward water and indomethacin and also supports the elevated dissipation values at high hydration in the presence of UCA.

The high-frequency impedance data showed close to 90° phase shift between the applied voltage and the resulting current. This indicates that the high-frequency impedance response for the lipid films is close to purely capacitive, with negligible contribution from resistive or other nonideal elements. In contrast, typical phase shifts obtained with excised skin membranes vary between 60 and 80°, illustrating the complex and nonideal capacitance components of the SC (44). The capacitance of the SC is intricately linked to its dielectric characteristics, which encompass the low-conductivity lipid matrix, lipid-protein domains, and charged lipid and protein species that contribute to double-layer capacitance (44,54,55). Typical values of the effective capacitance of excised skin membranes fall between 10 and 50 nF/cm^2^ (44,56). These values are around one order of magnitude higher compared with the values obtained with the lipid films (0.7–1.7 nF/cm^2^). This deviation implies that the capacitive domains of the SC are not only comprising the lipid matrix, which is exclusively probed here, but also other components of the SC. For instance, it has been previously suggested that capacitive currents, originating from double-layer charging, may be generated by lipid-protein domains or charged amino acid residues, for example, within the keratin filament (44,54).

Notably, the estimated dielectric constant increases when comparing the initial and final values, which can be explained by incorporation of water, with a dielectric constant of about 79, in the lipid films. However, both the absolute increase and dynamics of this hydration process differ between the samples (cf. Fig. 5 *b* where *C*_*eff*_ is proportional to *ε*). For UCA, the change occurs rapidly and to a lesser extent (40%), while for the glycerol sample, the change occurs more linearly resulting in a final increase of 143%. These observations suggest that the dielectric properties of the capacitive domains of the lipid film containing UCA are readily accessible for water and then remain constant, which is in line with more accessible void regions discussed above. The slower hydration process for the lipid film comprising glycerol indicates a more cohesive lipid matrix, which is supported by the observed higher proportion of orthorhombic chain packing. Thus, slower hydration dynamics is expected assuming that glycerol forms hydrogen bonds with the polar lipid headgroup and that the hydration process requires breaking and making of new hydrogen bonds with water molecules.

The observed effects of glycerol presented here should be compared with the extensive research that has explored how glycerol interacts with various lipid types, such as phospholipids and glycosphingolipids, particularly in its role as a cryoprotectant. When comparing glycerol’s effects on these different lipid classes, including the more nonpolar SC lipids investigated in this study, several differences and similarities emerge. For example, compression isotherms have shown that glycerol causes a slight expansion of monolayers of DPPC, DOPC, POPC, and POPE, indicating that glycerol integrates into the headgroup regions, regardless of the phospholipid species (57). In contrast, compression isotherms for glycosphingolipids monolayers showed the opposite effect (58), suggesting that the structural properties of the lipid headgroup significantly influence glycerol’s impact on the overall molecular organization. Notably, molecular dynamics simulations revealed that glycerol has a strong ability to form hydrogen bonds on facing leaflets of DPPC bilayers, creating a denser and more cohesive solvent layer between the phospholipid bilayers (59). In line with this suggestion, another study concluded that glycerol enhances the intermolecular cohesion of water molecules in the headgroup-solvent interfacial region, based on diffusivity measurements of water at the surface of DPPC bilayers, a property correlated with the hydrated volume of lipid headgroups (60). In summary, these findings indicate that glycerol and water interact with lipid headgroups in a comparable manner, while the effective outcome of these interactions may vary depending on the precise lipid headgroup architecture.

### Role of glycerol and UCA in the lipid matrix of the epidermal barrier

The observed effects of UCA can be related to the (patho)physiological processes in the upper layers of the SC, where the intercellular lipids are indeed less ordered than in the middle layers of the SC (61), and the processes necessary for desquamation are activated (62). It is possible that UCA, by contributing to lipid loosening and elevated hydration of the intercellular space, is one of the factors that allows adequate activation of the protease system that degrades corneodesmosomes (63). Consistent with this hypothesis, inadequate desquamation has been described in mice or humans with mutations leading to loss of filaggrin and hence UCA (44,53). Another aspect related to UCA is the fact that this substance undergoes *cis-trans* isomerization upon exposure to sun light. If we assume that the isomerization of *trans*-UCA to *cis*-UCA (27) also takes place to some extent in the lipid matrix, the localization of this molecule in a less rigid environment also makes sense. Consequently, the presence of UCA may result in less cohesive regions or leaflets in the lipid matrix upon hydration while leaving the acyl chain-rich domains essentially unchanged. This selective reorganization may lead to slipping under shear stress induced by the oscillating quartz crystal, which could potentially explain the significant increase in dissipation at high hydration without any significant structural alterations. Consequently, such UCA-induced regions of a less cohesive lipid matrix could enable higher diffusion rates for water, electrolytes, and the model substance.

Glycerol, on the other hand, is expected to be distributed more homogeneously throughout the SC lipid matrix, compared with UCA, as it is transported from below by aquaporin-3 (29) and from above by the degradation of surface lipids (30). Therefore, glycerol’s inability to fluidize and permeabilize the barrier lipids (although so far only valid for our in vitro system with 5 mol % glycerol) seems to make sense, as otherwise the lipid barrier would be compromised. Thus, the overall unchanged molecular structure and functional properties of the SC lipids in the presence of glycerol suggests that it is confined within lipid layers, enhancing the stability and cohesion of the lipid matrix through hydrogen bonds with polar lipid heads, rather than acting as a humectant. This proposed mechanism may be associated with previous observations in aquaporin-3-deficient mice (29) and asebia J1 mice (30), showing that skin barrier abnormalities are corrected by glycerol but not by water or urea. Although the effect of glycerol on skin lipids described in this study was not pronounced, it must be pointed out that 5 mol % glycerol means one glycerol molecule per roughly 20 lipid molecules and its native concentration may be higher; for example, water is present at 1–2 molecules per lipid. Thus, glycerol may be one important piece of the puzzle that leads to a competent epidermal barrier.

## Data and code availability

Data are available from the authors upon request.

## Acknowledgments

This study was supported by the 10.13039/501100001824Czech Science Foundation project no. 22-20839K (to I.S., G.P., A.K., and K.V.), 10.13039/100007397Charles University (SVV 260 661, to I.S. and I.H.), by the project New Technologies for Translational Research in Pharmaceutical Sciences/NETPHARM, ID CZ.02.01.01/00/22_008/0004607, cofunded by the 10.13039/501100000780European Union (to K.V., G.P., and A.K.), and by the research network Biobarriers – Health, Disorders and Healing, funded by the Swedish Knowledge Foundation (to M.M., E.J.N., and S.B., grant no. 20190010).

## Author contributions

Conceptualization, S.B. and K.V.; data curation, I.S., M.M., E.J.N., S.B., and K.V.; funding acquisition, K.V. and I.S.; investigation, I.S., M.M., G.P., E.J.N., I.H., A.K., S.B., and K.V.; methodology, I.S., M.M., G.P., E.J.N., I.H., A.K., S.B., and K.V.; supervision, S.B. and K.V.; validation, I.S., M.M., G.P., E.J.N., and S.B.; visualization, all authors; writing – original draft, I.S., S.B., and K.V.; writing – review & editing, all authors.

## Declaration of interests

The authors declare no competing interests.
